# A multilocus assay reveals high nucleotide diversity and limited differentiation among Scandinavian willow grouse (*Lagopus lagopus*)

**DOI:** 10.1186/1471-2156-9-89

**Published:** 2008-12-19

**Authors:** Sofia Berlin, Maria Quintela, Jacob Höglund

**Affiliations:** 1 Department of Evolutionary Biology, Evolutionary Biology Centre, Uppsala University, Norbyvägen 18 D, 752 36 Uppsala, Sweden; 2 Department of Plant Biology and Forest Genetics, Swedish Agricultural University, Ulls väg 24 E, 756 51 Uppsala, Sweden; 3 Department of Population Biology, Evolutionary Biology Centre, Uppsala University, Norbyvägen 18 D, 752 36 Uppsala, Sweden

## Abstract

**Background:**

There is so far very little data on autosomal nucleotide diversity in birds, except for data from the domesticated chicken and some passerines species. Estimates of nucleotide diversity reported so far in birds have been high (~10^-3^) and a likely explanation for this is the generally higher effective population sizes compared to mammals. In this study, the level of nucleotide diversity has been examined in the willow grouse, a non-domesticated bird species from the order Galliformes, which also holds the chicken. The willow grouse (*Lagopus lagopus*) has an almost circumpolar distribution but is absent from Greenland and the north Atlantic islands. It primarily inhabits tundra, forest edge habitats and sub-alpine vegetation. Willow grouse are hunted throughout its range, and regionally it is a game bird of great cultural and economical importance.

**Results:**

We sequenced 18 autosomal protein coding loci from approximately 15–18 individuals per population. We found a total of 127 SNP's, which corresponds to 1 SNP every 51 bp. 26 SNP's were amino acid replacement substitutions. Total nucleotide diversity (*π*_*t*_) was between 1.30 × 10^-4 ^and 7.66 × 10^-3 ^(average *π*_*t *_= 2.72 × 10^-3 ^± 2.06 × 10^-3^) and silent nucleotide diversity varied between 4.20 × 10^-4^and 2.76 × 10^-2 ^(average *π*_*S *_= 9.22 × 10^-3 ^± 7.43 × 10^-4^). The synonymous diversity is approximately 20 times higher than in humans and two times higher than in chicken. Non-synonymous diversity was on average 18 times lower than the synonymous diversity and varied between 0 and 4.90 × 10^-3 ^(average *π*_*a *_= 5.08 × 10^-4 ^± 7.43 × 10^3^), which suggest that purifying selection is strong in these genes. *F*_ST _values based on synonymous SNP's varied between -5.60 × 10^-4 ^and 0.20 among loci and revealed low levels of differentiation among the four localities, with an overall value of *F*_ST _= 0.03 (95% CI: 0.006 – 0.057) over 60 unlinked loci. Non-synonymous SNP's gave similar results. Low levels of linkage disequilibrium were observed within genes, with an average r^2 ^= 0.084 ± 0.110, which is expected for a large outbred population with no population differentiation. The mean per site per generation recombination parameter (ρ) was comparably high (0.028 ± 0.018), indicating high recombination rates in these genes.

**Conclusion:**

We found unusually high levels of nucleotide diversity in the Scandinavian willow grouse as well as very little population structure among localities with up to 1647 km distance. There are also low levels of linkage disequilibrium within the genes and the population recombination rate is high, which is indicative of an old panmictic population, where recombination has had time to break up any haplotype blocks. The non-synonymous nucleotide diversity is low compared with the silent, which is in agreement with effective purifying selection, possibly due to the large effective population size.

## Background

Until now, knowledge of the amount of nucleotide diversity in birds has been restricted to the domesticated chicken [1], although a few passerine species have been the subject of nucleotide diversity studies, e.g. red-winged blackbird [2], collared and pied flycatchers, blue tits and great reed warblers [3,4]. Estimates of nucleotide diversity reported so far in birds have been high (~10^-3^), approximately one order of magnitude higher than in for instance humans [5]. A likely explanation for this is the generally higher effective population sizes compared to mammals. This reasoning is based on that silent nucleotide diversity, *π*_*S *_= 4 *N*_*eμ*_, is directly dependent on effective population size (*N*_*e*_) and the mutation rate per generation (*μ*), and that *μ *is expected to be about the same in birds and mammals [6]. An alternative explanation for variation in nucleotide diversity could be differences in the occurrence of selective sweeps [7]. The latter suggests a relationship between the rate of recombination and the level of genetic diversity, as has indeed been shown for example in *Drosophila *[8] and humans [9,10]. This is particularly obvious in genomic regions with limited or no recombination, e.g. the female specific and non-recombining W chromosome in birds, that have unusually low levels of nucleotide diversity [11] as well as mammalian Y chromosomes that also harbour low levels of nucleotide diversity [5,12,13]. In order to understand variation in nucleotide diversity within genomes and between different lineages, accurate assessment of variation in recombination rates is required.

Single nucleotide polymorphisms (SNP's) are increasingly utilised as genetic markers for inferences of population genetic processes and molecular studies in ecology and evolution [12-15]. The use of SNP's has several advantages compared to more traditional markers such as microsatellites that commonly suffer from severe problems with homoplasy, null alleles and variable mutation patterns. Furthermore, the strong tradition of using mitochondrial DNA (mtDNA) is also seriously limited by the fact that it is a single, maternally inherited locus, and particularly in birds, likely subject to various forms of selection [14]. Like microsatellites, mtDNA has also a very high incidence of homoplasy when distances among taxa increase [15]. Although SNP's contain less information than both microsatellites and mtDNA, they are abundant in most species and evolve in a manner well described by simple mutation models (infinite sites model). A useful way of discovering SNP's in non-model organisms is by designing primers in exons in a species with considerable genomic data to amplify the corresponding exon (or intron) in a species with limited genetic data (i.e. the EPIC approach, [20,21]). This method has for example been used to amplify 242 introns in several diverse avian species by designing primers in conserved regions between the chicken genome sequence and orthologous zebra finch sequences [3].

In this study, the level of nucleotide diversity has been examined in the willow grouse, a non-domesticated bird species from the order Galliformes, which also holds the chicken. We collected samples from four locations in Scandinavia and used SNP's in 18 different autosomal exons located on different chromosomes in the genome. In addition to genetic diversity analyses, we estimated local levels of linkage disequilibrium (LD) and ρ, the population recombination rate. We also looked for population differentiation among the four sampling locations.

The willow grouse (*Lagopus lagopus*) has an almost circumpolar distribution but is absent from Greenland and the north Atlantic islands. It primarily inhabits tundra, forest edge habitats and sub-alpine vegetation. In Scandinavia, where the distribution of willow grouse overlaps with the closely related rock ptarmigan (*Lagopus mutus*), they generally occur at lower elevations and in wetter habitats with denser vegetation than the rock ptarmigan [16]. They breed in early summer and can produce up to two clutches per year, with on average eight eggs per clutch [17]. Willow grouse are hunted throughout its range, and regionally it is a game bird of great cultural and economical importance.

## Results

### Nucleotide variation in willow grouse

**Figure 1:**
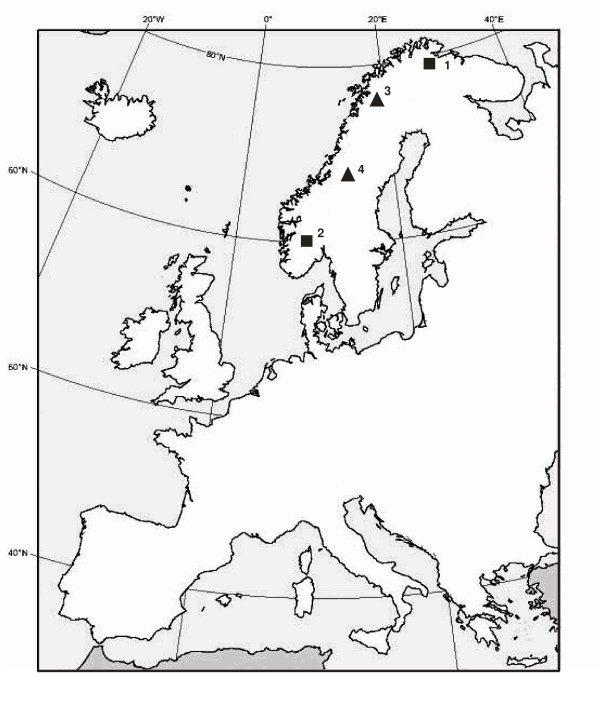
**Map of sampling localities**. The willow grouse samples were collected in 1. Nesseby, 2. Hardangervidda, 3. Tjuoltadalen and 4. Tjalling.

Sequences were obtained for 18 loci in 64 individuals; 15 from Tjuoltadalen, 18 from Tjalling, 15 from Hardangervidda and 16 from Nesseby (Figure 1). A total of 6571 bp (excluding gaps) were aligned over the 18 genes, resulting in a total of approximately 385 kb of sequence information across individuals.

**Figure 2:**
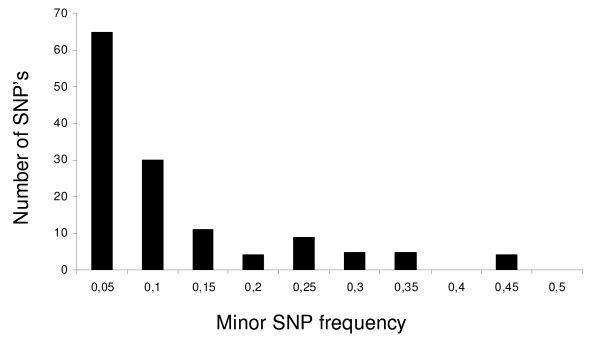
**SNP frequency spectrum combined across genes**.

We identified a total of 127 segregating sites, of which 32 were singletons and 95 parsimony informative. This corresponds to 1 SNP every 51 bp. Clearly, the frequency spectrum is skewed towards rare frequency variants (Figure 2). One parsimony informative site was found with three variants (*EPN2*) and one with all four possible variants (*MICRO*). 26 SNP's were amino acid replacement substitutions. Statistics of sequence variation are summarized in Table 1 and 2. Total nucleotide diversity (*π*_*t*_) was between 1.30 × 10^-4 ^and 7.66 × 10^-3 ^(average *π*_*t *_= 2.72 × 10^-3 ^± 2.06 × 10^-3^) and silent nucleotide diversity varied between 4.20 × 10^-4 ^and 2.76 × 10^-2 ^(average *π*_*S *_= 9.22 × 10^-3 ^± 7.43 × 10^-4^). Non-synonymous diversity was on average 18 times lower than the synonymous diversity and varied between 0 and 4.90 × 10^-3 ^(average *π*_*a *_= 5.08 × 10^-4 ^± 7.43 × 10^-3^).

**Table 1:** Total nucleotide variation in 18 autosomal loci in willow grouse.

Gene	L (gaps excl)	S (sing)	Theta (t)	Pi (t)
*AKR*	468	3 (1)	0.00121	0.00033
*APOA*	345	3 (0)	0.00167	0.0024
*BCL-2*	442	25 (7)	0.01076	0.00766
*BCL-X*	492	4 (4)	0.00151	0.00013
*BRIP1*	304	12 (3)	0.00746	0.00485
*CAAX*	437	9 (0)	0.00381	0.00357
*CXCR4*	291	5 (1)	0.00327	0.00204
*EPN2*	376	3 (1)	0.0015	0.00161
*KELCH*	443	20 (4)	0.0084	0.00551
*LEPR*	221	5 (2)	0.00437	0.00251
*MBL*	451	3 (0)	0.00128	0.00256
*MICRO*	279	8 (0)	0.00558	0.00609
*NGF*	324	4 (1)	0.00233	0.00234
*PKP4*	222	4 (2)	0.00335	0.0023
*PPARG*	295	3 (0)	0.0019	0.00135
*TAR*	467	3 (1)	0.00119	0.00101
*TRANS*	336	4 (0)	0.00222	0.00171
*YTH*	378	9 (5)	0.0046	0.00107

Total	6575	127	0.0037 ± 0.0028	0.0027 ± 0.0021

**Table 2:** Nucleotide variation for non-synonymous and synonymous sites separately.

Gene	L (nonsyn)	S (nonsyn)	Theta (a)	Pi (a)	L (syn)	S (syn)	Theta (s)	Pi (s)
*AKR*	349	1	0.00054	0.00029	116	2	0.00325	0.00046
*APOA*	262	0	0	0	83	3	0.00696	0.01
*BCL-2*	324	5	0.00294	0.00045	117	20	0.0324	0.02758
*BCL-X*	378	1	0.00049	0.00004	114	3	0.00488	0.00042
*BRIP1*	237	9	0.00719	0.0049	66	3	0.00854	0.00475
*CAAX*	329	3	0.00169	0.00074	106	6	0.01047	0.01241
*CXCR4*	219	1	0.00087	0.00033	69	4	0.01098	0.00752
*EPN2*	288	0	0	0	87	4	0.00869	0.00698
*KELCH*	337	0	0	0	104	20	0.03565	0.02341
*LEPR*	166	4	0.00465	0.00215	53	1	0.00365	0.00374
*MBL*	341	0	0	0	106	3	0.00542	0.01101
*MICRO*	212	0	0	0	64	6	0.01835	0.01946
*NGF*	247	1	0.00077	0.00014	74	3	0.00766	0.00978
*PKP4*	151	1	0.00123	0.00011	68	3	0.00822	0.00727
*PPARG*	225	0	0	0	69	3	0.00815	0.0058
*TAR*	363	0	0	0	102	3	0.00543	0.0046
*TRANS*	239	0	0	0	91	4	0.00823	0.0064
*YTH*	282	0	0	0	93	9	0.01869	0.00436

Total	4949	26	0.0011 ± 0.0020	0.00051 ± 0.0012	1582	100	0.011 ± 0.0092	0.0092 ± 0.0074

No gene had a statistically significant Tajima's D value [18], but the average value over all genes was negative, (-0.52 ± 0.85), indicating that an excess of low frequency variants are segregating in the population (Figure 2). Similarly, Fay and Wu's H tests [19] gave no statistically significant results for any locus, but the average H across loci was negative (-1.34 ± 2.12) (Table 3). The average synonymous sequence divergence (K_S_) between chicken and willow grouse was 0.13 ± 0.05 and the average non-synonymous sequence divergence (K_A_) was 0.015 ± 0.023 (Table 3). There is a particularly high variation in K_A _among the genes, which either suggests that selective pressure varies between different domains of the protein and/or among these genes.

**Table 3:** Neutrality tests in 18 willow grouse loci and pairwise Ks and Ka estimated with chicken orthologues.

Gene	D	H	K_S_	K_A_	K_A_/K_S_
*AKR*	-1.28	0.15	0.052	0.0085	0.16
*APOA*	0.78	-1.39	0.100	0.012	0.11
*BCL-2*	-0.85	-0.78	0.099	0.010	0.10
*BCL-X*	-1.75	0.06	0.080	0.0058	0.072
*BRIP1*	-0.91	-5.1	0.094	0.044	0.47
*CAAX*	-0.15	-2.15	0.15	0.0057	0.038
*CXCR4*	-0.79	-0.61	0.11	0.0014	0.013
*EPN2*	-0.38	0.55	0.09	0	*n.a.*
*KELCH*	-0.97	-2.61	0.25	0.041	0.17
*LEPR*	-0.91	-1.09	0.18	0.09	0.51
*MBL*	1.80	-0.10	0.16	0	*n.a.*
*MICRO*	-0.33	0.32	0.17	0.0052	0.030
*NGF*	0.0091	-0.19	0.084	0.0051	0.061
*PKP4*	-0.6	0.29	0.14	0.0083	0.059
*PPARG*	-0.5	-3.2	0.12	0.0036	0.029
*TAR*	-0.26	0.36	0.14	0.018	0.13
*TRANS*	-0.45	-1.24	0.17	0.0063	0.037
*YTH*	-1.91	-7.41	0.11	0.0051	0.046

Total	-0.52 ± 0.85	-1.31 ± 2.12	0.13 ± 0.047	0.015 ± 0.023	0.11 ± 0.15

### Linkage Disequilibrium

Generally, low levels of linkage disequilibrium were observed within genes, with an average r^2 ^= 0.087 ± 0.12. D' within genes was on average < 1. For two genes, (*BRIP1 *and *TRANS*), r^2 ^was higher than 0.30 (D' < 1). The per site per generation recombination parameter (ρ) was estimated for the 16 genes with more than two segregating sites (*BCL-X*, *EPN2 *and *TAR *had less than two and were not analysed). Of the 15 loci, only four produced 95% CI's within ρ = 0 – 100. Maximum likelihood estimates of ρ per site was 0.045 in *BRIP1*, 0.043 in *CXCR4*, 0.015 in *PKP4 *and 0.010 in *TRANS*, with a mean of 0.028 ± 0.018.

### Population Structure

*F*_ST _values based on synonymous SNP's varied between -5.60 × 10^-4 ^and 0.20 among loci and revealed low levels of differentiation among the four populations, with an overall value of *F*_ST _= 0.03 (95% CI: 0.006 – 0.057) over 60 unlinked loci. Pairwise *F*_ST_'s varied between 0.005 to 0.045, and three out of six comparisons were statistically significant (Table 4). *F*_ST _values based on non-synonymous SNP's varied between -0.005 and 0.233 over 12 loci, with an overall *F*_ST _= 0.049 (95% CI: -0.019 – 0.121). None of these 60 unlinked synonymous and nonsynonymous SNP's show a significant excess of heterozygote's. The program STRUCTURE revealed the highest likelihood for K = 3 for both test statistics ((LnP(D) and ΔK).

**Table 4:** Geographical coordinates.

	Nesseby	Tjuoltadalen	Tjalling	Hardangervidda
Nesseby (Norway) (70° 08' N; 29° 00' E)		0.00513 (p > 0.05)	0.04549 (p = 0.001)	0.04247 (p = 0.021)
Tjuoltadalen (Sweden) (67° 27' N; 17° 14' E)	552		0.00546 (p > 0.05)	0.02459 (p > 0.05)
Tjalling (Sweden) (63° 6' N; 12° 26' E)	1084	580		0.04107 (p = 0.004)
Hardangervidda (Norway) (58° 37' N; 7° 25' E)	1647	1141	564	

Below diagonal, distances in km between localities and above diagonal, pairwise Fst with p-values in parentheses.

### Confirmation of SNP's and PHASE haplotypes by cloning

The cloned sequences were compared with the directly sequenced PCR products for each individual and marker. In total, 48 SNP's were compared between cloned and directly sequenced PCR products. In one case, a heterozygote had been interpreted as a homozygote in the directly sequenced product, which means that heterozygotes were correctly assigned based on direct sequencing of PCR products in 98% of the cases. In addition, we compared the haplotypes based on the PHASE assignments with the results from the cloning experiment. The clones containing PCR products from the three individuals for marker *BCL-2*, yielded six different haplotypes, the three individuals cloned for marker *CAAX *produced five different haplotypes, the three individuals cloned for marker *KELCH *also produced five different haplotypes, while the two individuals cloned for *TRANS *produced two different haplotypes. All SNP's except one were correctly assigned in haplotypes.

## Discussion

Polymorphism levels in non-domesticated bird species have been very little investigated, apart from in a few passerine species. Particularly, extensive SNP surveys in wild close relatives to the avian model organism, the chicken are lacking. We used the chicken genome as template for primers and amplified the orthologous regions in the willow grouse. We amplified 18 exons in individuals from four populations in Scandinavia. Levels of nucleotide diversity in coding regions of willow grouse (*π*_*t *_= 2.72 × 10^-3^) was substantially higher than in humans [5] and higher than in many other mammalian species [20-22]. The synonymous nucleotide diversity is particularly high (*π*_*S *_= 9.22 × 10^-3^), especially compared to the non-synonymous diversity (*π*_*a *_= 5.08 × 10^-4^). The low ratio between *π*_*a *_and *π*_*S *_(*π*_*a*_/*π*_*S *_= 0.055) indicate that that new mutations entering the populations are effectively selected against. In no bird species so far have such high silent polymorphism levels been reported. What population genetic processes most likely explain this pattern? Theory holds that silent nucleotide diversity is dependent on the mutation rate per generation and the effective population size. There are reports of very large numbers of breeding pairs of willow grouse in Norway and Sweden, and figures of more than one million have been reported . If this corresponds with a large N_e _such a large effective population sizes are comparable with those of *Drosophila melanogaster*, where similarly high levels of nucleotide diversity have been observed [23]. Estimates of neutrality (Tajima's D and Fay and Wu's H) revealed no evidence of selection in any of the genes. However, mean Tajima's D was negative (-0.52 ± 0.85) as was mean Fay and Wu's H (-1.34 ± 2.12), which is indicative of low frequency variants segregating in the populations. This could be an effect of population expansion since the retreat of the ice around 10.000 years ago, followed by re-colonisation [24].

There was a significant but weak population differentiation among the four locations (*F*_ST _= 0.03, 95% CI: 0.006 – 0.057) and a positive but non-significant correlation between genetic and geographic distances. The program STRUCTURE estimated the most likely number of populations to three, however the biological meaning of this result is questionable as the likelihood for K = 1, K = 3 and K = 4 are very similar. The low levels of differentiation are most likely explained by the large number of birds in Scandinavia and a more or less continuous habitat. From an extensive study where 300 individuals were captured and equipped with radio-transmitters [16] female natal dispersal was estimated at 10.2 km, while males on average dispersed three times shorter distances (3.4 km). In Norway natal dispersal was estimated to be 4.2 km for both sexes combined [25]. These distances are longer than for the majority of forest and plain grouse [16]. Effective dispersal distance in hazel grouse in northern Sweden was estimated to 1.4 km using microsatellites [26] and significant local population structure was detected in black grouse males but not females [27]. Local structure has also been detected in Scottish red grouse (*Lagopus lagopus scoticus*) using both mtDNA and microsatellite loci [31,32]. However, previous studies of population structure among Scandinavian willow grouse populations using allozymes found low degrees of genetic differentiation [28-30].

One remarkable result of the current study is the extremely low levels of linkage disequilibrium found in the willow grouse (mean r^2 ^= 0.087 ± 0.12, mean D' < 1). It is possible that the pattern we observe is an effect of demographic factors rather than effects of recombination events. In a large population (stable or expanding), accumulation of SNPs can lead to low correlation (r^2^) between neighbouring SNPs even in the absence of recombination. In such cases, D' as a measure of LD should be much more informative as it tends to be a more accurate reflection of the historical association between two loci. The fact that we within exons always have some pairwise D' < 1 suggest that recombination can have caused the low r^2 ^values that we observe. Obviously, in order to fully understand patterns of LD in the willow grouse genome we need many more markers located in different regions of the genome. Studies of LD in other avian species, particularly in natural populations are rare. LD in domesticated chicken have been estimated for two genomic regions in three different commercial breeds with different levels of inbreeding and large variations in LD was found both between breeds as well as between genomic regions [31].

In contrast to the low levels of LD between autosomal markers in willow grouse, extensive LD was found among closely located markers on the Z chromosome in the passerine species collared flycatchers [32]. A difference in LD between autosomes and the Z chromosome is interesting and implies that there might be selection for a reduction in recombination on Z. A recent study using collared and pied flycatchers as model organisms have shown that species recognition has a genetic component [33] and other studies have shown that disproportionately many genes that are involved in reproductive isolation are located on Z [49,50]. Taken together, these observations suggest that Z chromosomes in birds are enriched for speciation genes.

Linkage mapping in species belonging to the order Galliformes, suggest high recombination rates compared to mammals. Groenen et al. estimated the chicken linkage map length to 3800 cM [34] and although the physical size of the chicken genome is threefold smaller than most mammalian genomes [35], the genetic maps are of similar sizes. Similarly, both turkey and quail have long linkage maps compared to their genome sizes [36-38]. Whole-genome linkage mapping in birds outside the Galliformes order is in its infancy, although recently two linkage maps were published for species belonging to the order Passeriformes, the zebra finch [39], and the collared flycatcher [40]. Both maps were shorter than the chicken map, despite supposedly similar genome sizes.

Population based approaches for estimating recombination is an attractive alternative to linkage mapping, since it can be difficult to obtain well defined pedigrees. One such way of studying recombination is by investigating the per generation population recombination rate (ρ), which is a function of both crossing-over frequency and effective population size. This has been estimated in red winged blackbirds and ρ ranged between 0.045 to 0.225 [2]. In the willow grouse we estimated mean ρ to 0.028 ± 0.018 in four genes. This again suggests that birds exhibit much higher rates of recombination in general than for example humans [41] and seem more similar to *Drosophila *[42]. Again, a likely explanation that the per generation recombination rate is high in birds, is that they generally have large effective population sizes. This fits very well with the high levels of silent nucleotide diversity seen in birds.

## Conclusion

Scandinavian willow grouse have an unusually high silent nucleotide diversity compared with other species. There is very little population structure among the four geographically distant locations. This suggests that the effective population size is high, but possibly also that the mutation rate is high. The non-synonymous nucleotide diversity is low compared with the silent, which is in agreement with effective purifying selection, possibly due to the large effective population size. The population shows signs of population expansion, which probably dates back to the last glaciations. There is very little linkage disequilibrium within the genes and the population recombination rate is high, which is indicative of an old panmictic population, where recombination has had time to break up any haplotype blocks.

## Methods

### Tissue samples and Sampling

Wings or breast muscle from 64 shot willow grouse were collected at two localities in Norway; Nesseby (70° 08' N; 29° 00' E) (16 individuals) and Hardangervidda (58° 37' N; 7° 25' E) (15 individuals) and in Sweden; Tjuoltadalen (67° 27' N; 17° 14' E) (15 individuals) and Tjalling (63° 6' N; 12° 26' E) (18 individuals), respectively (Figure 1 and Table 4). These four locations span the north-south distribution of willow grouse in Scandinavia. DNA was extracted from small biopsies (a few mm^3^) using a salt extraction protocol.

### Primer design, PCR and Sequencing

Primers were designed based on annotated chicken protein coding genes in Genbank  (Table 5). In order to generate the largest possible number of base pairs per sequencing reaction, the major criterion for choosing a locus was that it had to have an exon length exceeding 500 bp. We also wanted to target as many different chromosomes as possible based on the chicken karyotype, as there are three different chromosomal classes (micro, intermediate and macro), each possessing distinct features such as gene content, recombination rate and GC content [35], which potentially could affect polymorphism levels. See Table 6 for marker information and primer sequences. PCR-amplifications were performed in 15 *μ*l reactions in an Applied Biosystems Gene Amp PCR Systems 2700 thermal cycler. Individual mixes contained approximately 40 ng DNA template, 1 × PCR buffer, 1.5–2.5 mM MgCl_2_, 1 × GC, 0.1 mM dNTP, 0.25 *μ*M of each primer, and 0.375 U FastStart Taq Roche polymerase (See also Table 6). PCR profiles consisted of 5 min denaturation at 95°C followed by 33–40 cycles of 35s denaturation at 95°C, 45 s annealing at 50–55°C and 1 min extension at 72°C with a final 5 min 72°C step (See also Table 6). To avoid contamination, DNA extractions, pre PCR and post PCR pipetting were carried out in different rooms and aerosol-resistant filter pipette tips were used throughout. The PCR products treated with ExoSAP-IT (USB Corporation) before sequencing reactions were ran with Dyenamic ET terminators (GE Healthcare, Piscataway, NJ). All products were sequenced on a MegaBace 1000 capillary instrument (GE Healthcare, Piscataway, NJ). Individuals from the two Norwegian populations were sequenced both ways. For all loci, except *CXCR4*, *LEPR *and *MBL*, the sequence quality was so high that the remaining samples were sequenced using the forward primer only. Sequences were edited in Sequencher v. 4.6 (Gene Codes).

**Table 5:** Information on which genes that were amplified together with GenBank accession numbers and position in the chicken genome (from the UCSC Genome Browser).

Gene	*Gallus gallus *gene name	Accession number	Position in chicken genome (chromosome: nucleotide pos.)	Exon	Exon length (base pairs)
*AKR*	homeodomain protein AKR	U25353	chr2:103.921.346-103.921.912	2	567
*APOA*	apolipoprotein A-I	M17961	chr24:5.237.113-5.237.710	2	598
*BCL-2*	bcl-2	D11382	chr2:69.060.949-69.061.501	1	553
*BCL-X*	bcl-x	Z23110	chr20:9.982.309-9.982.859	one exon	551
*BRIP1*	BRCA1 interacting protein C-terminal helicase 1	NM_001033058	chr19:7.487.075-7.487.928	19	854
*CAAX*	phosphatase 1 regulatory (inhibitor) subunit 16B	NM_001030851	chr20:3.798.908-3.799.425	9	518
*CXCR4*	chemokine (C-X-C motif) receptor 4	NM_204617	chr7:32.377.230-32.378.282	one exon	1053
*EPN2*	epsin 2	NM_001012788	chr14:5.218.405-5.218.997	1	593
*KELCH*	similar to kelch-like 10	XM_418155	chr27:4.334.591-4.335.186	3	596
*LEPR*	leptin receptor	NM_204323	chr8:29.155.374-29.156.177	18	804
*MBL*	mannan-binding lectin associated serine protease 3	AY567829	chr9:15.984.978-15.985.841	10	864
*MICRO*	microfibrillar-associated protein 3 (MFAP3)	NM_001012784	chr13:12.338.307-12.339.000	2	694
*NGF*	nerve growth factor	XM_418016	chr26:3.859.235-3.859.771	one exon	537
*PKP4*	plakophilin 4	NM_001006529	chr7:38.046.479-38.047.037	6	559
*PPARG*	peroxisome proliferative activated receptor gamma	NM_001001460	chr12:5.081.300-5.081.735	5	436
*TAR*	TAR DNA binding protein	NM_001030878	chr21:4.069.862-4.070.391	1	530
*TRANS*	UbiA prenyl-transferase domain containing 1	NM_001030879	chr21:4.196.588*-4.197.005	1	502
*YTH*	YTH domain family member 1	NM_001012833	chr20:8.697.322-8.698.208	4	887

*The first 84 nucleotides were denoted as N's in the genome sequence

**Table 6:** Primer sequences and PCR conditions.

**Gene**	**Primer sequences**	**Step lengths (s) (annealing/extension)**	**MgCl_2 _(Mm)**	**No. of cycles**	**T_a _(°C)**
*AKR*	F: GTCTGCAACTGGTTTATCAAC R: TTAGGCCATGAGTTTTGCCTG	30/45	1.5	33	50
*APOA*	F: CCTGAAGCTGGCTGACAACC R: TCAGGCCACGGACTTCTGG	30/45	1.5	38	50
*BCL-2*	F: GAGAAGAGGCTACGACAACC R: CCATCCTCCGTTGTCCTGG	30/45	1.5	33	50
*BCL-X*	F: ATGTCCAGCAGTAACCGGG R: CAGCCGCCATTCTCCTGG	30/45	1.5	33	50
*BRIP1*	F: GTGGCCAGAAAGTTGATGTAG R: CTATGTGCTAGTACTGTCCAG	45/60	2.5	40	50
*CAAX*	F: CACTGCTGGAGAGTTCTTCC R: CTAGGAAATCCTGCAGCAC	45/60	1.5	35	55
*CXCR4*	F: TCCTCTGGCATACTCATTG R: GCTGGAATGGAAACTTGAAG	45/60	2.5	40	50
*EPN2*	F: GACGACTTCATCTATTAGGCG R: AGCCATGGTAGGATGCTGGAG	45/60	2.5	40	50
*KELCH*	F: CAGACCACTTCATGAACAAAG R: CTTATCATGCAGCGTGGTTG	45/60	2.5	40	50
*LEPR*	F: CCTGAAACTTTTGAGCAYC R: TTAACAGCTGTTCTCTGTGG	45/60	2.5	40	50
*MBL*	F: ATCATCAAGCGCATCATCGG R: TCATCTCTCCAGCTCAGGG	45/60	2.5	40	50
*MICRO*	F: GTGGACGGTGGAGGATTGCTG R: GTGTCAATGCTCTGCGCTTC	45/60	2.5	40	50
*NGF*	F: ATGCCAGATGGAACAGAAG R: TCAGGGTCTCCCCGATTTC	30/45	1.5	38	50
*PKP4*	F: CCTTCAGCAAACGTTGCTAC R: TGTAAGGCTATCTGGACGTG	45/60	2.5	40	50
*PPARG*	F: GACATGAACTCTTTAAGGATGG R: CTCCACTTAGTATAATGACAGC	45/60	2.5	40	50
*TAR*	F: TTGCCCAGTCTCTTTGTGGAG R: CTACATTCCCCAGCCTGA	45/60	2.5	40	50
*TRANS*	F: GAGCTGGTGCAGAAGATCAG R: CTCCGGTATAGAGGAAGGAG	45/60	2.5	40	50
*YTH*	F: GTGAAGCGCCATGGTCTAC R: GGACTCACCACTTGGAACAG	45/60	2.5	40	50

### Nucleotide Diversity and Divergence

Estimates of standard population genetics parameters and neutrality test statistics were calculated for each locus with DnaSP v. 4.10.9 [43]. Insertions and deletions are reported, but not included in any analyses. Pairwise sequence divergence between chicken and willow grouse was estimated as K_A _and K_S _using the Nei-Gojobori algorithm [44] with Jukes-Cantor correction [45] in MEGA 3.1 [46].

### Haplotype Phasing

Haplotype phasing was performed using PHASE v. 2.1 [40,41] on all parsimony informative SNP's for each gene separately.

### Linkage Disequilibrium and Population recombination rate

The level of linkage disequilibrium (LD) between parsimony informative sites within genes was estimated in Haploview [47], both as r^2^, the mean squared correlation in allelic state between pairs of SNP's and D', the difference between the observed and the expected gamete frequencies standardised by the theoretical maximum for the observed allele frequencies. These two estimates of LD are somewhat different and it has been argued that r^2 ^has more power to detect lack of recombination than D' [48]. D' = 1 means complete LD and occurs if at least one allele at each locus is completely associated with an allele at the other locus, however D' < 1 is somewhat difficult to interpret. Both estimates are considered to be allele frequency dependent, but r^2^, less so than D'. Phased data was used in these analyses. The population recombination parameter ρ = 4N_e_r was estimated using Ldhat 2.0 [49], where N_e _is the effective population size and r is the per site per generation recombination rate. Likelihoods were obtained for recombination rates in the range, ρ = 0 – 100 and 95% CI was calculated.

### Population Structure

Population differentiation was estimated using Weir & Cockerhams F_ST _statistic [50] in Genetix 4.03 [51]. Significance was assessed from 1000 permutations. Synonymous and non-synonymous SNP's were analyzed separately. All analyses were carried out on a subset of the data represented by only parsimony informative unlinked loci. We used a LOD score cutoff of 3 to infer linkage. LOD scores were estimated for each pair of SNP's in Haploview [47].

The genetic structure of Scandinavian willow grouse was also investigated with the model-based clustering algorithm implemented in STRUCTURE v. 2.1 [52,53]. We used the admixture model, with population information, five runs with a burn-in of 100,000 and a run length of 1,000,000 for a number of clusters from K = 1 to K = 10, allowing for correlation of allele frequencies between clusters. We performed five independent runs per K to ensure that the results were consistent. We investigated the most likely number of clusters in two different ways, by considering the log 'probability of data' lnP(D) for the different numbers of K, and by using the statistic ΔK (Evanno et al), which considers the rate of change in lnP(D) among successive K values. Hardy-Weinberg exact tests for heterozygote excess was performed in Genepop on the web using option 1.

Geographical distances between populations were estimated in kilometers (km) using Google Earth.

### Confirmation of SNP's and PHASE haplotypes by cloning

Cloning of PCR products from four markers, *BCL-2*, *CAAX*, *KELCH *and *TRANS *was performed using pGEM^®^-T Easy Vector System II following the manufacturer's protocols (Promega). PCR products from three individuals were cloned for *BCL-2*, *CAAX*, *KELCH *and two for *TRANS*. For each marker, eight colonies were amplified with illustra TempliPhi Amplification Kit (GE Healthcare) and finally sequenced by Macrogen Inc. (Macrogen, Seoul, South Korea) on ABI 3730 instruments (Applied Biosystems).

## Authors' contributions

SB designed the primers, analysed the data and wrote the manuscript. MQ carried out all lab work. JH designed and coordinated the study. All authors read and approved the final manuscript.
